# Methodical control of the difficult pediatric airway: two case reports

**DOI:** 10.1186/s13256-023-03788-2

**Published:** 2023-03-08

**Authors:** A. Low, D. Hunter, H. A. Baboolal

**Affiliations:** 1 Department of Anesthesiology, University of North Carolina, 101 Manning Drive, Chapel Hill, NC 27514 USA; 2 University of North Carolina, Chapel Hill, USA; 3 Department of Anesthesiology, University of North Carolina, Chapel Hill, USA

**Keywords:** Airway: Pediatric versus adult, Pediatrics: Airway management, Upper airway anatomy

## Abstract

**Background:**

Management of children who present with a history of impossible mask ventilation or difficult tracheal intubation is fraught with challenges. Despite this, the “airway stress test” of an inhalational induction is frequently employed risking airway obstruction, breath holding, apnea, and laryngospasm.

**Case presentations:**

We present two cases of children with anticipated difficult airway management. The first child (14-year-old African American boy) had severe mucopolysaccharidosis with a history of failed anesthetic induction and failed airway management. The second child (3-year-old African American girl) had progressive lymphatic infiltration of the tongue, resulting in severe macroglossia. We describe a technique that forgoes inhalational induction, incorporates recent pediatric airway guidelines, and provides a greater margin of safety. The technique encompasses the use of drugs that facilitate sedation for intravenous access, without respiratory depression or airway obstruction, titrated use of medications to achieve anesthetic depth while preserving ventilatory drive and airway tone, and the continuous provision of directed oxygen flow during airway manipulation. Propofol and volatile gases were avoided to preserve airway tone and respiratory drive.

**Conclusions:**

We emphasize that an intravenous induction technique utilizing medications that preserve airway tone and ventilatory drive, and the use of  continuous oxygen flow throughout airway manipulation, allows for successful management of children with a difficult airway. The common practice of volatile inhalational induction should be avoided in anticipated difficult pediatric airways.

## Introduction

The difficult pediatric airway poses unique challenges, in contrast with its adult counterpart. The original American Society of Anesthesiologists difficult airway guidelines were  not specifically directed toward the pediatric patient, and did not address safe induction practices in this population [1]. The Society for Pediatric Anesthesia has not provided a formalized pathway, but has offered guidance via the Pediatric Difficult Intubation registry. A special issue of Pediatric Anesthesia was recently published, dedicated to the difficult pediatric airway and provided some direction in the cases we report below [2].

We describe two cases of children with challenging airways and offer a framework for approaching this issue in a careful step wise fashion. We contrast how the choice of induction technique can have a major impact resulting in either success or a failed airway. We address inhalational versus intravenous induction, and the selection of drugs to provide optimal conditions. The emphasis in these reports is on a safe induction approach, maintaining spontaneous ventilation, preservation of airway tone, and continuous oxygenation. This manuscript adheres to the Anesthesia Case Report (ACRE) reporting guidelines.

## Report

Case 1: A 14-year-old, 42 kg African American boy with severe mucopolysaccharidosis type II (Hunter syndrome) and worsening upper airway obstruction presented for elective tracheostomy. He presented 4 months previously for bronchoscopy under general anesthesia. At the initial presentation he was receiving noninvasive ventilator support by face mask at night and had cognitive delay and moderate mitral regurgitation. During the previous anesthetic, he underwent a failed inhalational anesthetic induction with sevoflurane, during which the patient became increasingly difficult to mask ventilate. Urgent flexible fiberoptic bronchoscopic attempts to secure the airway failed, and a laryngeal mask was placed. Ventilation via the laryngeal mask was unsuccessful and further attempts to intubate fiberoptically via the laryngeal mask failed. The decision was made to abort the procedure. The child regained consciousness without further complications and was discharged home.

In the interim, he continued to receive noninvasive ventilator support by face mask at night. The child also began to request ventilator support during the daytime. He was scheduled for tracheostomy due to worsening upper airway obstruction.

At presentation, his airway examination was notable for a short neck, minimal neck extension, macroglossia, and a Mallampati class IV. Considering the previous failed inhalational induction, we decided to place an intravenous catheter after premedication, and perform a stepwise intravenous induction while maintaining spontaneous respiration. He received 3.5 ml of 4% nebulized lignocaine, and 4% lignocaine cream was applied to his thigh. After 20 minutes he received intramuscular dexmedetomidine 0.3 µg kg^−1^, ketamine 1.2 mg kg^−1^, and glycopyrrolate 10 µg kg^−1^. Within 30 minutes, he was slightly sedated without signs of airway obstruction. While he was watching a movie, it took several attempts to secure intravenous access, during which he was calm and cooperative.

**Fig. 1:**
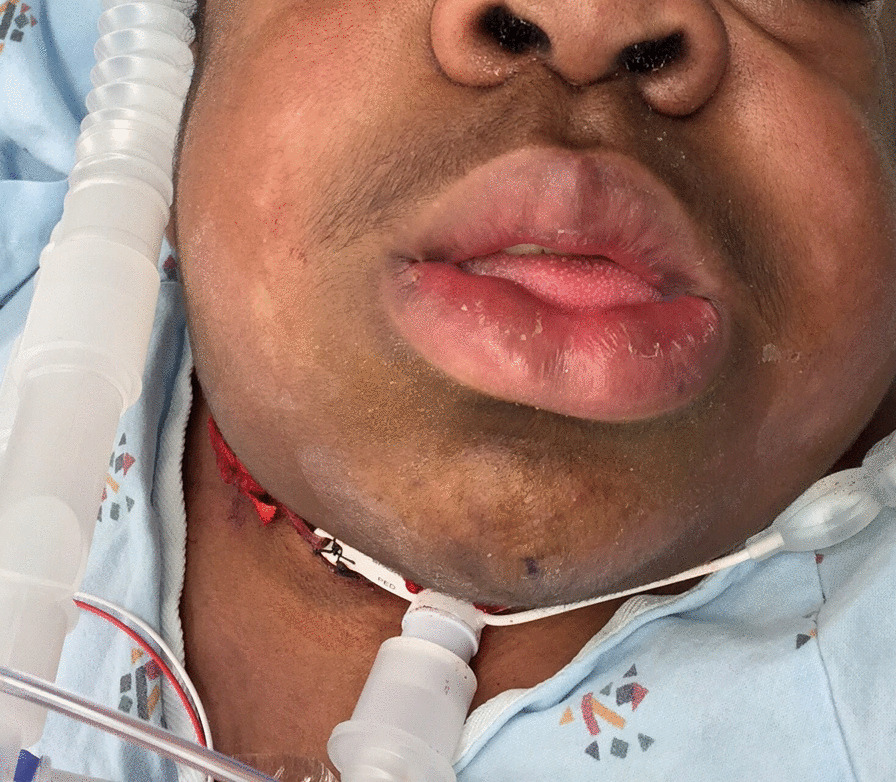
Child with Hunter syndrome demonstrating severe macroglossia and short neck

Intravenous induction commenced in the sitting position with incremental doses of ketamine (total dose 2 mg kg^−1^) and dexmedetomidine (0.3 µg kg^−1^) over 10 minutes, while breathing oxygen by face mask. As anesthetic depth progressed, a mask seal was established, and intermittent mask ventilation was successful without airway devices. A 24 French nasopharyngeal airway was inserted in his nose and connected to the anesthesia circuit via a tracheal tube connector to provide continuous oxygenation at 10 l min^−1^. Oral flexible fiberoptic tracheal intubation was achieved in the setting of spontaneous ventilation. There was initial difficulty in identifying the glottic inlet, due to soft tissue deposits and mucosal edema of the epiglottis and arytenoids. The vocal cords were finally identified due to phasic movement during respiration. There were no significant episodes of oxygen desaturation or hemodynamic issues during airway instrumentation. Tracheostomy proceeded uneventfully (Fig. 1) over 90 minutes, although with some difficulty, due to anatomic distortion.

**Fig. 2:**
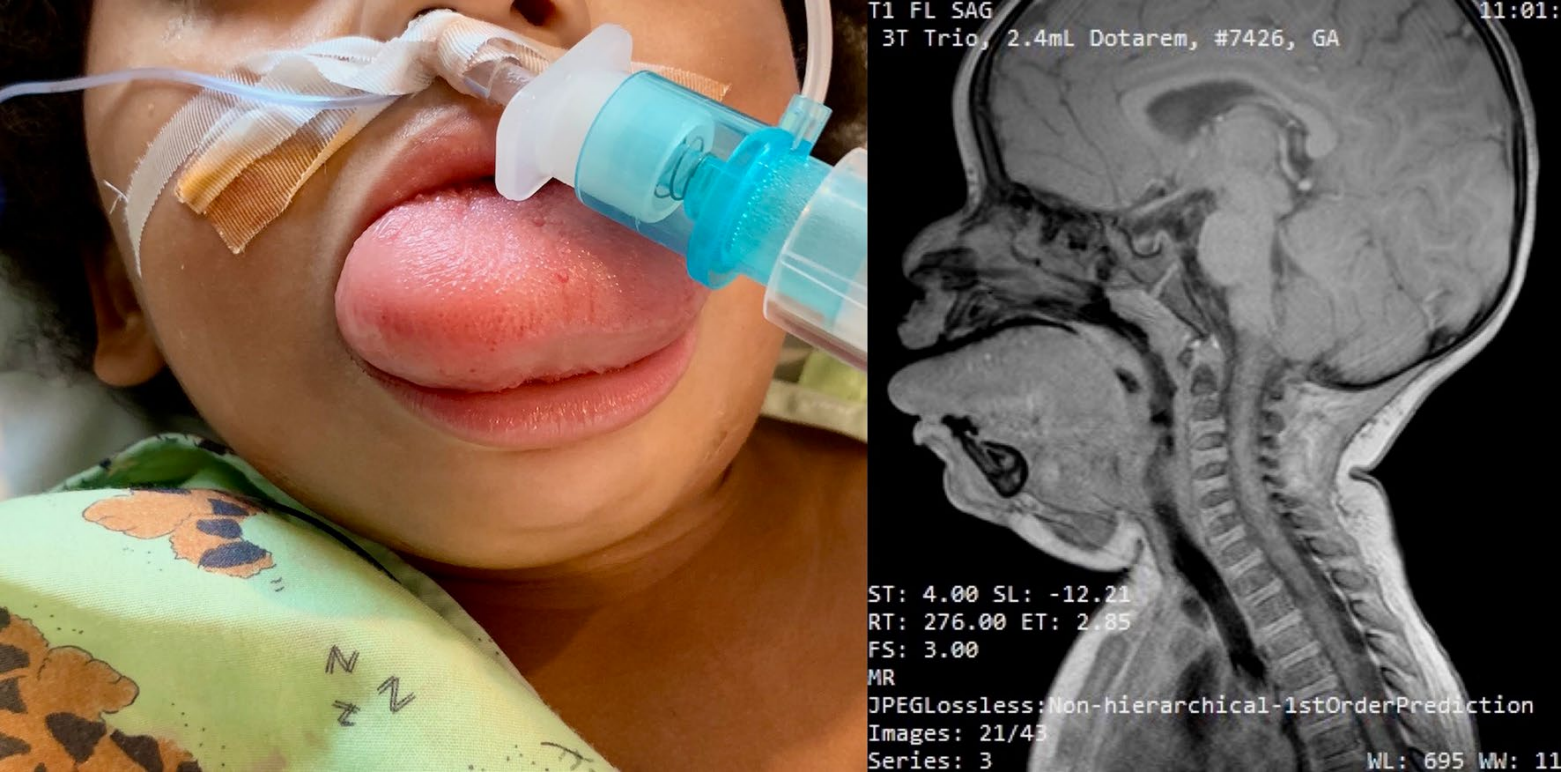
Child with lymphatic tongue malformation demonstrating severe macroglossia, and magnetic resonance imaging demonstrating soft tissue lymphatic infiltration

Case 2: A 3-year-old, 19 kg African American girl with progressive enlargement of a lymphatic tongue malformation presented for sclerotherapy (Fig. 2). She demonstrated severe macroglossia and limited mouth opening. Magnetic resonance imaging (MRI) scan showed infiltration of the sublingual area, submandibular, parotid, and parapharyngeal spaces (Fig. 2). A concentration of 4% lignocaine cream was placed on her thigh and intramuscular ketamine 4 mg kg^−1^ was given to facilitate intravenous catheter placement. She then received incremental doses of intravenous ketamine (total 1.5 mg kg^−1^) and glycopyrrolate (10 µg kg^−1^). A nasopharyngeal airway with tracheal tube connector was placed in the left nostril and connected to the anesthesia circuit to provide supplemental oxygen. Pressure support ventilation was initiated while we successfully performed nasal fiberoptic tracheal intubation via the contralateral nostril. Spontaneous ventilation, hemodynamic stability, and oxygenation were maintained throughout the induction and intubation.

## Discussion

In 2016 the USA Pediatric Difficult Intubation Registry published data from 13 children’s hospitals in the USA, aiming to identify intubation practices and complications in 1018 pediatric patients with difficult tracheal intubation [3]. Approximately 80% of patients were anticipated difficult airways. Despite the anticipation of difficult intubation, 64% of patients underwent inhalational induction. However, 8% proved to be difficult to mask ventilate after induction.

Inhalational induction could arguably be labeled as an airway stress test, due to its narrow and unpredictable therapeutic index, with risk of breath-holding, obstruction, laryngospasm, and apnea [4]. The history of an aborted inhalational induction in our first case demonstrates that a “mask” induction in a child with a difficult airway can quickly result in difficulty maintaining mask ventilation, and that a supraglottic airway may not serve adequately as a rescue device. In addition, emergent tracheostomy may be challenging in the setting of severe anatomical airway distortion. The sobering incidence of difficult mask ventilation in this population emphasizes the point that the time is ripe for an alternative approach to induction in these children.

We have described the management of two children with challenging airway anatomy, one of whom had a previous anesthetic with failed airway management. Traditional anesthetic induction in the pediatric population employs inhalational agents, due to the apparent safety of this technique and partly due to a perceived ability to maintain spontaneous respiration [5]. However, in these two cases we describe a cautious, controlled technique that involves preinduction intravenous access, adequate deep sedation with preserved ventilatory drive, and continuous and directed high-flow oxygen with a modified nasopharyngeal airway during airway manipulation.

In our cases, the patients had clinical features and history suggesting that both mask ventilation and intubation would be difficult or impossible. The patient with Hunter syndrome acted as his own case control with a previous history of failed mask ventilation, failed LMA placement, and failed flexible fiberoptic tracheal intubation. The potential inability to establish face mask ventilation eliminated a major pathway of the ASA difficult airway algorithm. In addition, awake fiberoptic intubation was not practical, due to age and cognitive development. As mentioned above, anesthesia in these patients are often induced by volatile agents, without establishing intravenous access. However, the ideal induction technique in this situation would provide deep sedation, while maintaining respiration and upper airway tone, and pose minimal risk of apnea or obstruction.

We achieved these goals by securing intravenous access after sedation with intramuscular ketamine and dexmedetomidine. This was followed by titrated intravenous sedation with ketamine and dexmedetomidine, keeping in mind the twin goals of maintaining spontaneous respiration and upper airway tone. Propofol was avoided, due to its effect on upper airway tone [6]. Following recent guidelines, continuous directed oxygen flow was achieved during airway manipulation by attaching a nasopharyngeal airway to the anesthesia circuit via a 15 mm tracheal tube connector [7].

In addition to the safety aspect, spontaneous respiration also dispensed with the need to interrupt airway instrumentation to oxygenate our patients. It potentially limited the risk of moderate/severe hypercarbia seen in apneic oxygenation. Ketamine and dexmedetomidine were valuable due to their synergistic ability to provide sedation and cooperation for intravenous access, and subsequently for their ability to allow airway instrumentation while maintaining tone in the airway structures and avoiding the risk of airway obstruction [8]. In addition, this technique provided an unexpected advantage in the child with Hunter syndrome, because spontaneous vocal cord movement helped to identify the larynx in a distorted anatomical field.

In 2020, Pediatric Anesthesia published a special issue dedicated to management of the difficult airway. The editorial entitled “Learning, Unlearning and Relearning” called for a cognitive shift in the approach to the pediatric airway, and in particular the difficult airway [9]. Within that context, our case reports offer a paradigm shift, away from the perceived safety of inhalational anesthetic induction in the child with a difficult airway, and toward a technique that allows for a greater margin of safety.

Barriers to routine adoption of intravenous induction in the difficult pediatric airway include cultural institutional practice, patient and parental anxiety and distress, the potential for difficult intravenous access, and lack of familiarity in placing intravenous catheters in an awake child.

## Conclusion

We have described a technique of methodical, titrated intravenous induction in two pediatric patients with a history of failed or difficult airways, which provides superior conditions to traditional volatile anesthetic inhalational induction. The choice of medications preserved respiratory drive and airway tone, facilitating intravenous access in a calm patient and titration to provide adequate depth of anesthesia during airway manipulation. In combination with continuous supplementary oxygen flow, this offers a potentially safer approach than the widespread practice of volatile inhalational induction.

## Data Availability

Data described in this article is available upon request from the authors, and is anonymized to protect patient identity.
